# Antibiofilm and antivirulence potential of silver nanoparticles against multidrug-resistant *Acinetobacter baumannii*

**DOI:** 10.1038/s41598-021-90208-4

**Published:** 2021-05-24

**Authors:** Helal F. Hetta, Israa M. S. Al-Kadmy, Saba Saadoon Khazaal, Suhad Abbas, Ahmed Suhail, Mohamed A. El-Mokhtar, Noura H. Abd Ellah, Esraa A. Ahmed, Rasha B. Abd-ellatief, Eman A. El-Masry, Gaber El-Saber Batiha, Azza A. Elkady, Nahed A. Mohamed, Abdelazeem M. Algammal

**Affiliations:** 1 Department of Medical Microbiology and Immunology, Faculty of Medicine, Assiut University, Assiut, 71515 Egypt; 2 Department of Internal Medicine, University of Cincinnati College of Medicine, Cincinnati, OH 45267-0595 USA; 3 Branch of Biotechnology, Department of Biology, College of Science, AL-Mustansiriyah University, POX 10244, Baghdad, Iraq; 4 Faculty of Science and Engineering, School of Engineering, University of Plymouth, Plymouth, PL4 8AA UK; 5 Wolfson Nanomaterials and Devices Laboratory, School of Computing, Electronics and Mathematics, Faculty of Science and Engineering, Plymouth University, Devon, PL4 8AA UK; 6 Department of Pharmaceutics, Faculty of Pharmacy, Assiut University, Assiut, 71526 Egypt; 7 Department of Pharmacology, Faculty of Medicine, Assiut University, Assiut, 71515 Egypt; 8 Centre of Excellence in Environmental Studies (CEES), King Abdulaziz University, Jeddah, 21589 Saudi Arabia; 9 Microbiology and Immunology Unit, Department of Pathology, College of Medicine, Jouf University, Al-Jouf, Saudi Arabia; 10 Department of Medical Microbiology and Immunology, College of Medicine, Menoufia University, Menoufia, Egypt; 11 Department of Pharmacology and Therapeutics, Faculty of Veterinary Medicines, Damanhour University, Damanhûr, 22511 Egypt; 12 Sohag University Medical Administration, Sohag University, Sohâg, 82524 Egypt; 13 Department of Medical Biochemistry, Faculty of Medicine, Assiut University, Assiut, 71515 Egypt; 14 Department of Bacteriology, Immunology, and Mycology, Faculty of Veterinary Medicine, Suez Canal University, Ismailia, 41522 Egypt

**Keywords:** Microbiology, Pathogenesis

## Abstract

We aimed to isolate *Acinetobacter baumannii* (*A. baumannii*) from wound infections, determine their resistance and virulence profile, and assess the impact of Silver nanoparticles (AgNPs) on the bacterial growth, virulence and biofilm-related gene expression. AgNPs were synthesized and characterized using TEM, XRD and FTIR spectroscopy. *A. baumannii* (n = 200) were isolated and identified. Resistance pattern was determined and virulence genes (*afa/draBC, cnf1, cnf2, csgA, cvaC, fimH, fyuA, ibeA, iutA, kpsMT II, PAI, papC, PapG II, III, sfa/focDE* and *traT)* were screened using PCR. Biofilm formation was evaluated using Microtiter plate method. Then, the antimicrobial activity of AgNPs was evaluated by the well-diffusion method, growth kinetics and MIC determination. Inhibition of biofilm formation and the ability to disperse biofilms in exposure to AgNPs were evaluated. The effect of AgNPs on the expression of virulence and biofilm-related genes (*bap, OmpA, abaI, csuA/B, A1S_2091, A1S_1510, A1S_0690, A1S_0114*) were estimated using QRT-PCR. In vitro infection model for analyzing the antibacterial activity of AgNPs was done using a co-culture infection model of *A. baumannii* with human fibroblast skin cell line HFF-1 or Vero cell lines. *A. baumannii* had high level of resistance to antibiotics. Most of the isolates harbored the *fimH*, *afa/draBC*, *cnf1*, *csgA* and *cnf2,* and the majority of *A. baumannii* produced strong biofilms. AgNPs inhibited the growth of *A. baumannii* efficiently with MIC ranging from 4 to 25 µg/ml. *A. baumannii* showed a reduced growth rate in the presence of AgNPs. The inhibitory activity and the anti-biofilm activity of AgNPs were more pronounced against the weak biofilm producers. Moreover, AgNPs decreased the expression of *kpsMII* , *afa/draBC,bap, OmpA,* and *csuA/B* genes. The in vitro infection model revealed a significant antibacterial activity of AgNPs against extracellular and intracellular *A. baumannii*. AgNPs highly interrupted bacterial multiplication and biofilm formation. AgNPs downregulated the transcription level of important virulence and biofilm-related genes. Our findings provide an additional step towards understanding the mechanisms by which sliver nanoparticles interfere with the microbial spread and persistence.

## Introduction

*Acinetobacter* species are commonly found in the environment, however, some species within this genus are generally associated with various habitats such as soil, water, sewage, human, foods and animals. *Acinetobacter baumannii* (*A. baumannii*) has an inevitable ability to sustain harsh conditions and spread as a serious pathogen^1–4^. *A. baumannii* infects moist tissues such as mucous membranes or areas of the skin that are exposed to this pathogen, either through wound or injury^5,6^.

The adhesion and colonization or biofilm formation include primary stage in bacterial infections. Major adhesion virulence factors in this step include type I fimbriae (*FimH*) and pilli structures for attachment to the host cells^7,8^. Furthermore, numerous bacteria secrete toxins and extracellular enzymes which play a crucial role in the apoptosis or necrosis of epithelial cells or immunocytes. Various virulence factors of *A. baumannii* such as adhesins genes like *kpsMII* (group 2 capsule synthesis) and *fimH*, *tratT* (serum resistance associated), *fyuA* (yersiniabactin receptor) and *iutA* (aerobactin receptor) have been investigated previously^9,10^. An important polysaccharide for biofilm formation is encoded by *pgaABCD* locus^11^. Biofilm production is a strategy to escape from harsh conditions and immune responses, hence play as reservoirs for drug-resistant systemic infections. Biofilm-producing *A. baumannii* has been isolated from several infectious origins such as pneumonia and devise-associated infections. Bacterial within biofilm can resist significantly more against antibiotics compared to planktonic mode of growth^12^. Hence, biofilm-mediated infections are in relapse more frequently^13^.

Therefore, there is an urgent need to enhance the effects of antimicrobials against pathogenic bacteria. In recent years, interest has enhanced towards application of nanoparticles as therapeutic regimens^14–21^. Silver nanoparticles (AgNPs), which have low toxicity in ecosystems and have high rate of surface capacity, can inhibit accumulation of biofilm materials responsible for evasion and protection^22–24^.

The aim of this study was to isolate *A. baumannii* from wound infections, determine their resistance and virulence profile, and assess the impact of AgNPs on the bacterial growth, virulence and biofilm-related gene expressions in the isolated strains.

## Methods

### Ethical statements for human/animal experiments

The study was approved by the Institutional Review Board and ethics committee of Assiut University, Egypt and Mustansiriyah University, Baghdad, Iraq. A written informed consent was obtained from participants. All experiments were performed in accordance with relevant guidelines and regulations.

### Synthesis and characterization of silver nanoparticles (AgNPs)

AgNPs (1000 µg/ml) were prepared via chemical reduction of silver nitrate in presence of polyvinylpyrrolidone (PVP) as a stabilizer according to^25^. The obtained samples were stirred, forming AgNPs. AgNPs formation was proved by Ultraviolet–visible spectral analysis. The absorbance spectra were determined using UV spectroscopy at a wavelength of 300–700 nm. The obtained NPs were characterized using transmission electron microscopy (TEM), X-ray diffraction methods (XRD) and Fourier transform infrared (FTIR) spectroscopy^26,27^. In TEM, a drop of NPs dispersion was placed on a carbon-coated copper grid and dried at room temperature and the micrograph of the preparation was taken.

In case of XRD, crystalline structure of AgNPs powder was identified using the X-ray diffractometer with CuKα radiation (λ = 1.5405 Å) in the 2θ range of 10°–80°. For FTIR, samples were prepared by KBr-discs technique and the spectra were recorded in the range of 400–4000 cm^−1^.

### Bacterial isolates and identification of *A. baumannii*

Two hundred isolates of *A. baumannii* were identified from wound infections. Phenotypic identification included culture onto the MacConkey agar (OXOID, Basingstoke, England), Leed Acinetobacter agar (Hardy Diagnostics, Santa Maria, CA, USA), and CHROMagar Acinetobacter/MDR medium, and incubated under aerobic conditions at 37 C for 48 h. Gram staining, motility, oxidase, peroxidase, and oxidative-fermentation reactions were performed according to standard techniques. API 20NE bacterial identification system (Biomerieux, Marcy-l’Étoile, France) was used for species identification. Molecular identification of isolates was also conducted using PCR for detection of *recA* specific gene and *bla*_*-*oxa51_ gene as previously described^28^. The sequence of the primers are listed in supplemental Table 1.

### Antimicrobial susceptibility patterns

Following Clinical and Laboratory Standards Institute guidelines^29^, bacterial cultures were plated onto the surface of Muller Hinton agar plates (HiMedia Laboratories, Mumbai, India) and susceptibility was measured by the disc-diffusion methods. The following antibiotics were tested: cephalothin (30 μg), cotrimoxazole (23.75/1.25 μg), nitrofurantoin (300 μg), trimethoprim (5 µg), ceftazidime (30 µg), tobramycin (10 µg), amikacin (30 µg), tetracycline (30 μg), gentamicin (10 µg), streptomycin (10 µg), erythromycin (15 µg), levofloxacin (5 µg), rifampicin (5 μg), chloramphenicol (30 μg), azithromycin (15 µg), imipenem (10 μg) and ciprofloxacin (5 μg). *Staphylococcus aureus* ATCC25923, *A. baumannii* ATCC 19,606 and *Escherichia coli* ATCC25922 were used as quality control organisms in the test^29^.

### Screening for virulence-related genes

Virulence-related genes including *afa/draBC, cnf1, cnf2, csgA, cvaC, fimH, fyuA, ibeA, iutA, kpsMT II, PAI, papC, PapG II, III, sfa/focDE* and *traT* were screened using PCR. The primer sequence and product size are listed in Supplemental Table 1.

### Screening for the antibacterial activity of AgNPs

The antimicrobial activity of the AgNPs was evaluated by the well-diffusion method. *A. baumannii* cultures (containing about 10^5^ CFU/ml) were plated on the surface of Mueller Hinton agar plates (HiMedia Laboratories, India). Then, wells (5 mm is diameter) were made using a borer and 50 μg/ml of the AgNPs dispersions were added into the wells. Plates were then incubated at 37 °C for 24 h and the inhibition zone diameters were measured^30^.

### Determination of the minimum inhibitory concentration (MIC) of AgNPs

MIC of the formulated AgNPs against clinically isolated *A. baumannii* was determined using the broth micro-dilution method according to the guidelines of the Clinical and Laboratory Standards Institute^31^. Briefly, AgNPs formulation was serially diluted in 100 μl Mueller–Hinton broth in wells of 96-well plate. Bacterial suspensions were added and plates were incubated for 24 h at 37 °C. MIC was determined visually by determining the wells with no visible bacterial growth.

### Testing the biofilm strength using the microtiter plate method

The biofilm-forming abilities of the tested strains were carried out using the microtiter plate method as previously described^32^. Briefly, isolates were cultivated in Muller Hinton Broth (HiMedia Laboratories, India) containing 2% w/v sucrose (Sigma-Aldrich, USA) and incubated for 24 h at 37 °C in 96 well microtiter plates (1 × 10^5^ CFU/ml, 200 μl /well). After incubation, wells were gently washed twice with PBS solution. Biofilm formed by adherent organisms was stained with crystal violet (0.2% w/v) and the excess dye was washed with distilled water. After drying, 95% ethanol was added to the wells and the optical density (OD) was measured at 570 nm using a microplate reader. Average OD values were determined for all tested isolates and the negative controls. Cut-off value was defined as the value of mean OD of the negative control plus 3X standard deviation of the negative control reads. Isolates were considered weak biofilm producers if they have a mean OD values ≤ two times the cut-off value, moderate biofilm producer if OD ≤ four times the cut-off value and strong biofilm producer if OD > four times the cut-off value.

In some experiments, the effect of AgNPs on biofilm formation was tested. To do that, the isolates were treated with 100 µl of 25 µg/ml AgNPs in BHI medium and analyzed as mentioned before. The ability of AgNPs to reduce biofilm formation was assessed by comparing the OD of the AgNPs treated wells versus control untreated wells. To test the ability of AgNPs to disperse the pre-formed biofilm layers, bacterial suspensions were allowed to grow overnight in the 96-well plates, then different aliquots of AgNPs were added to the overnight bacterial cultures. Plates were incubated for additional 24 h, and finally, the strength of the biofilms exposed to the AgNPs was re-evaluated. The experiment was performed in triplicate for each isolate.

### Effect of AgNPs on microbial growth kinetics

Using the 96-well plate, we compared the effect of AgNPs on the growth of 3 representative *A. baumannii* strong, moderate and weak biofilm producers. Bacterial suspensions at OD595 in Muller Hinton broth were incubated at 37 °C in the presence of 5 μg/ml AgNPs (sub-MIC concentration) and OD595 was measured at 6, 24, 48 and 72 h using a microplate reader (Epoch, biotek, USA). Then, growth curves were plotted using the mean of three replicates for each strain ± SD^33^.

### Quantitative detection of the effect of AgNPs virulence and biofilm-related genes using RT-PCR

The effect of AgNPs (25 µg/ml) on the expression of the virulence-related genes (*kpsMII* and *afa/draBC* genes) and biofilm-related genes (*bap, OmpA, abaI, csuA/B, A1S_2091, A1S_1510, A1S_0690, A1S_0114*) was evaluated using RT-PCR. RNA was extracted from bacteria treated with AgNPs using TRIzol reagent (Sigma-Aldrich, Switzerland) and untreated bacteria were used as controls. cDNA was synthesized subsequently by Superscript III kit (Invitrogen Inc., USA) according to the manufacturer protocol. PCR reactions were carried out using ePower SYBR Green PCR Master Mix (Applied Biosystems, USA) in a 7500 Sequence Detection System (CFX 96 biorad, USA). Samples were normalized to the expression of the 16S rRNA gene and the fold change in the expression of the target genes was calculated using 2^−ΔΔCT^ method^34^. Expression level of each gene in AgNps treated cells was compared to the expression of untreated bacteria as a control.

### In vitro infection model for analyzing the antibacterial activity of AgNPs

The ability of AgNPs to kill extracellular and intracellular *A. baumannii* was determined using a co-culture infection model of *A. baumannii* and HFF or Vero cells, as described previously^35,36^. Briefly, 1 × 10^5^ HFF or Vero cells were seeded in a 24-well plate and incubated overnight at 37C with 5% CO_2_. Log phase bacterial suspension was prepared by incubating fresh colonies of *A. baumannii* overnight in Mueller–Hinton broth at 37C. Then, the bacterial suspension was diluted with broth and incubated for another 2 h at 37 C to reach the log phase of growth.

First, the extracellular killing activity was evaluated as previously described^35^. For this purpose, HFF or Vero cells were challenged with 2 × 10^5^ CFU/mL of *A. baumannii* then AgNPs (25 µg/ml) were added in a total volume of 0.5 mL. Control samples treated with PBS were included. Kinetic studies were conducted at different time points to evaluate the extracellular killing activity at different contact times (5, 15, 30 and 60 min). Post-treatment samples were serially diluted in sterile PBS, plated on Mueller–Hinton agar plates, incubated for 24 h at 37C and CFUs were counted. The extracellular killing activity of AgNPs was evaluated by determining the percentage killing at the different time points, which was calculated by the following formula: (CFU of the control untreated samples − CFU of the AgNPs treated samples/CFU of the untreated control samples) × 100.

Second, the ability of AgNPs to kill the intracellular *A. baumannii* was carried out using the same co-culture infection model of *A. baumannii* and human fibroblast skin cell line HFF-1or Vero cells^35^. Briefly, cells were challenged with 1 × 10^8^ CFU/mL log-phase *A. baumannii* and incubated for 2 h. Extracellular bacteria were removed by 2 h treatment with gentamycin then AgNPs (25 µg/ml ) or sterile PBS (plain control) were added and incubated for different time pointes (0, 2, 4, 8 and 16 h). Following incubation, cells were lysed by Triton X-100 (0.1%) and samples were diluted in PBS, plated on Mueller–Hinton agar plates, incubated for 24 h at 37C and CFUs were counted. The Intracellular killing activity of AgNPs was evaluated by determining the percentage killing at different time points relative to the control untreated cells as described previously. All experiments were performed in triplicate and the mean ± standard deviation was presented.

### Analysis of in vitro cytotoxicity of AgNPs

The cytocompatibility of AgNPs was evaluated using two cell lines; the human fibroblast skin cell line HFF-1 and Vero cell line (kindly provided by VACSERA tissue culture laboratory, Dokki, Giza, Egypt) as previously described^33^. About 1 × 10^4^ cells/200 ul were cultured in 96-well-plate at 37 C and 5% CO_2_ in DMEM (Gibco, Thermo Fisher Scientific, USA) supplemented with 10% Fetal Bovine Serum. After incubation of the cells overnight, AgNPs were added at different concentrations and incubated for 24 h. The viability of the fibroblast cells was measured by the MTT assay protocol (CellTiter 96 Non-Radioactive Cell Proliferation kit, Promega, USA) according to manufacturer instructions. Experiments were performed in triplicates and absorbance was measured at 570 nm using Epoch microplate reader (Bitek, USA).

### Statistical analysis

All the obtained results were statistically analysed with the GraphPad Prism (version 6.0, GraphPad, San Diego, CA, USA) and the statistical software package for the Social Sciences software (SPSS, version 16). A *p* value < 0.05 was considered statistically significant.

### Ethics approval and consent to participate

The study was approved by the Institutional Review Board and ethics committee of Assiut University, Egypt and Mustansiriyah University, Baghdad, Iraq. A written informed consent was obtained from participants. All experiments were performed in accordance with relevant guidelines and regulations.

## Results

### Characterization of synthesized AgNPs

**Figure 1:**
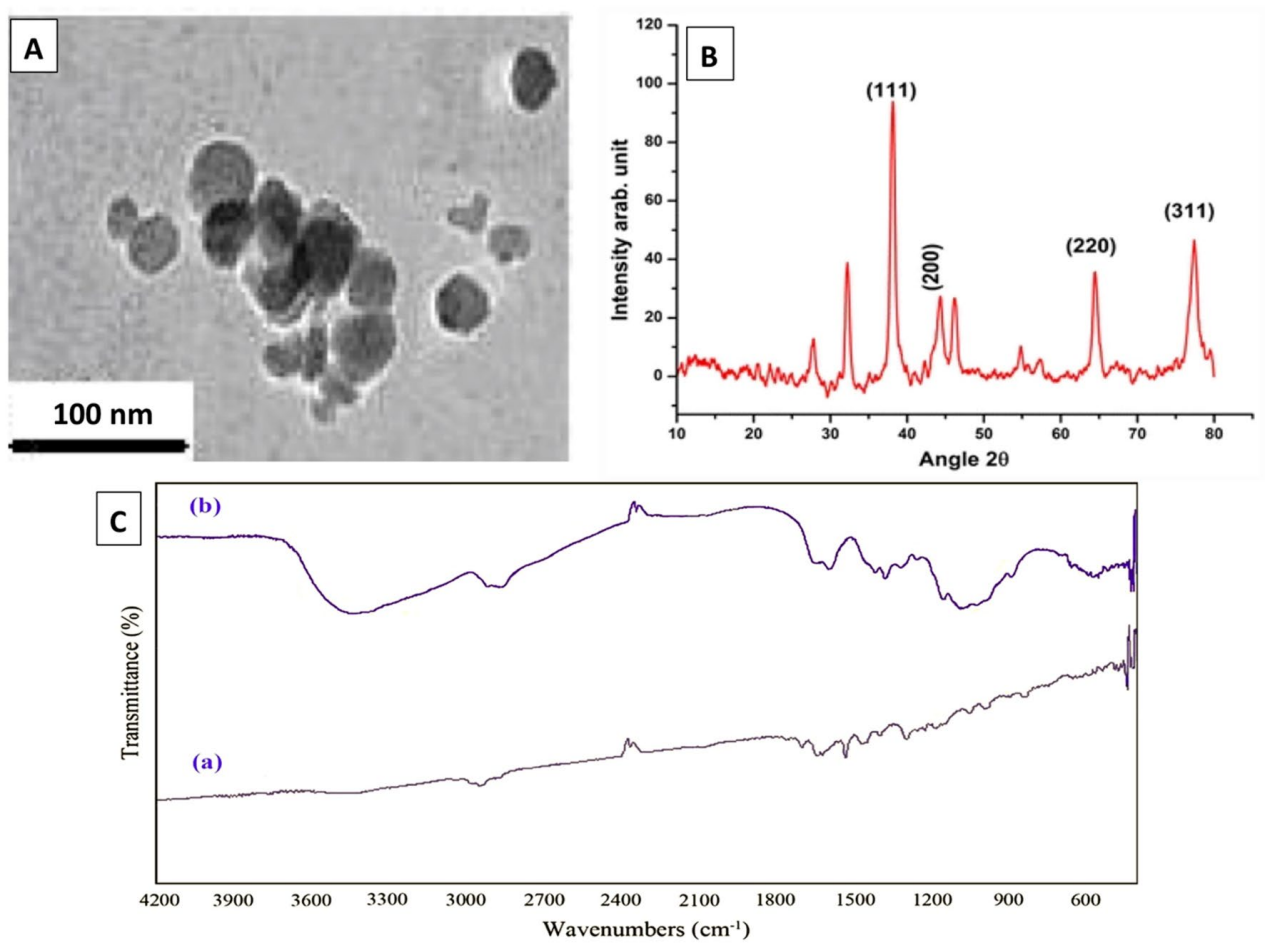
Characterization of synthesized silver nanoparticles (AgNPs). (**A**) The TEM image of AgNPs showed a spherical particles with size in nano range (10–50 nm, (**B**) X-ray diffraction analysis revealed the crystalline structure of AgNPs, (**C**) The FTIR analysis was done for AgNPs in the range of 400–4000 cm^−1^.

Shape and size of AgNPs, which were prepared via chemical reduction of Ag salts, were characterized using TEM. AgNPs had a spherical shape with particle size in the range of 10–50 nm (Fig. 1A). XRD measurement for a dried film from the concentrated suspension confirmed the crystalline structure of AgNPs (Fig. 1B). Figure 1C shows FTIR spectra for PVP and PVP/AgNPs. Spectrum of AgNPs stabilized with PVP was identical to that of pure PVP, showing PVP characteristic peaks at 1290 cm^−1^, 1670 cm^−1^ and 2900 cm^−1^, which correspond to CN bond, carbonyl group and C–H stretching, respectively^37,38^. Slight shifts in the spectrum of AgNPs indicate the formation of coordination bonds between oxygen or nitrogen of PVP with Ag atoms.

### Antibacterial susceptibility testing

Antibacterial susceptibility of the isolated *A. baumannii* isolates were tested against 17 antibiotics. These isolates were 100% resistant to 12 antibacterial agents (cephalothin, cotrimoxazole, trimethoprim, ceftazidime, tobramycin, amikacin, tetracycline, streptomycin, erythromycin, levofloxacin, chloramphenicol and ciprofloxacin). Only azithromycin, nitrofurantoin, rifampicin, imipenem and gentamycin displayed limited degrees of resistance. The resistance profile of the 200 A. *baumannii* isolates and distribution of the virulence-related genes are shown in supplemental file 2.

#### Evaluation of bacterial virulence profile

Most of the *A. baumannii* isolates (n = 200) carried the *fimH* (n = 160, 80%), *afa/draBC* (n = 146, 73%), *cnf1* (n = 112, 56%), *csgA* (n = 98, 49%) and *cnf2* (n = 86, 43%), followed by *ibeA* (n = 82, 41%), *cvaC* (n = 80, 40%), *iutA* (n = 80, 40%), *papC* (n = 78, 39%), *traT* (n = 77, 38.5%), *PAI* (n = 62, 31%), *fyuA* (n = 48, 24%), *kpsMII* (n = 47, 23.5%), *PapGII* (n = 34, 17%) and *papGIII* (n = 8, 4%). The majority of *A. baumannii* (n = 165, 82.5%) produced strong biofilms, 22 (11%) isolates produced moderate and 13 (6.5%) isolates produced weak biofilms.

#### Evaluation of the antibacterial and anti-biofilm activities of AgNPs

**Figure 2:**
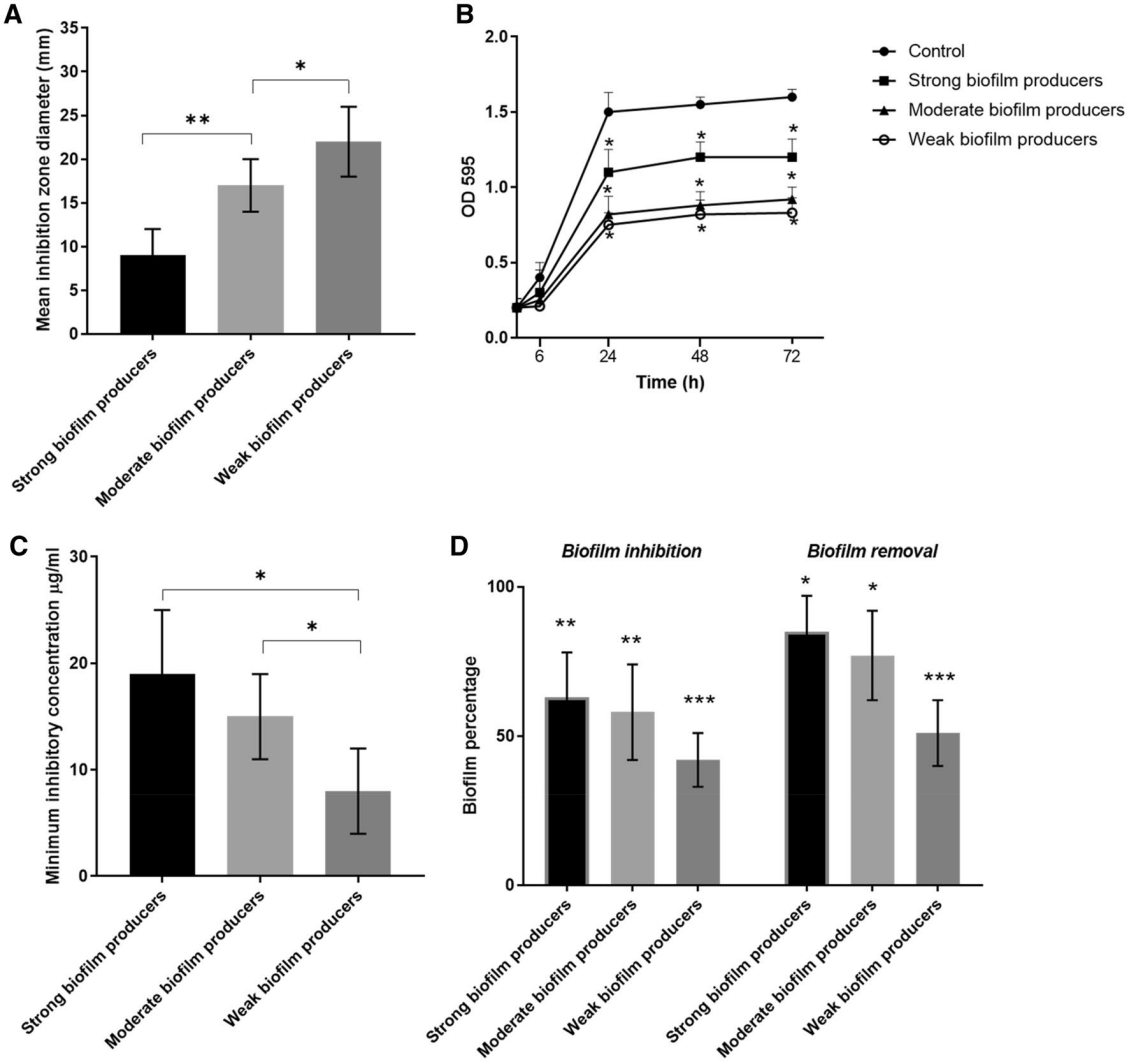
Antibacterial activity of AgNPs on the isolated *A*. *baumannii*. (**A**) Inhibition zone diameters induced by the silver nanoparticles. (**B**) Growth kinetics of 3 representative *A. baumannii* from each group in the presence of AgNPs. The microbial growth was estimated by the optical density (OD595). (**C**) MIC values according to strength of biofilm formation. (**D**) Effect of silver nanoparticles on biofilm inhibition and biofilm dispersion. Untreated bacteria were used as control. Columns show the mean ± SD. *p < 0.05 indicates statistical significance as compared to control by Student’s t-test.

Agar-well diffusion was used to test the inhibitory activity of AgNPs on bacterial growth. Exposure to 50 µg/mL AgNPs produced marked inhibition zones in all tested bacterial strains (mean = 16 mm and range = 6–27 mm). However, the biofilm formation seems to interfere with the formulation’s inhibitory activity. The inhibitory activity was more pronounced on weak biofilm producers, where AgNPs induced a mean inhibition zone diameter of 22 ± 5 mm, compared to 17 ± 4 mm and 10 ± 4 mm in moderate and strong *A. baumannii* biofilm producers (Fig. 2A).

The growth curve of *A. baumannii* strains was determined in the presence of silver nanoparticles or its absence as a control. The growth rate was estimated by measuring the OD595 at different time points (6, 24, 48 and 72 h). Normal cells showed a marked increase in OD595 after 6 h and reached a stationary phase over the duration of 24–72 h. Generally, *A. baumannii* showed a reduced growth rate in the presence of AgNPs (at 24, 48 and 72 h), compared to untreated cells. Consistent with the resistance phenotype of the strong biofilm producers, the effect was more obvious with moderate and weak biofilm producing bacteria (Fig. 2B).

A similar effect was observed when MIC values of the formulated AgNPs against the isolated *A. baumannii* were determined. MIC values of the AgNPs against *A. baumannii* were in the range of 4–25 µg/mL. Lowest MIC values were observed for the weak biofilm producers, followed by the moderate and strong biofilm forming isolates (8 ± 4 µg/mL, 15 ± 4 µg/mL and 19 ± 6 µg/mL, respectively). Significant difference was observed between strong and moderate biofilm producers vs the weak group as shown in Fig. 2C.

Furthermore, exposure to AgNPs inhibited the strong, moderate and weak biofilm production significantly and biofilms reached 63 ± 15%, 58 ± 16% and 42 ± 9% of controls. AgNPs were also able to disperse the already formed strong, moderate and weak biofilm. Percentage of biofilms treated with AgNPs were 85 ± 12%, 77 ± 15% and 51 ± 11%, respectively (Fig. 2D).

### Modulation of virulence and biofilm-related gene expression

The expression of *kpsMII* and *afa/draBC* genes were assessed with and without exposure to AgNPs. In presence of AgNPs, the expression of *kpsMII* and *afa/draBC* adhesin genes decreased by 4.6-folds (*p* = 0.001) and 3.4-folds (*p* = 0.001), respectively. RT-PCR analysis of total RNA isolated from strong biofilm producers treated with AgNPs showed that the transcription of *bap, OmpA, and csuA/B* was significantly decreased by 4.5-, 3.1- and 3.1-folds, respectively, when compared with the untrerated bacteria (Table 1). In contrast, the transcriptional expression of the *A1S_2091, A1S_1510, A1S_0690* and *A1S_0114* was not affected.

**Table 1:** Fold change in expression levels of virulence and biofilm-related genes in AgNPs-treated bacteria with respect to the untreated cells.

Target gene	Control untreated bacteria	AgNPs-treated bacteria	*p* value	Fold change
*kpsMII*	1 ± 0.156	0.22 ± 0.165	0.001*	4.6
*afa/draBC*	1 ± 0.234	0.29 ± 0.148	0.001*	3.4
bap	1 ± 0.335	0.23 ± 0.254	0.01*	4.5
*OmpA*	1 ± 0.017	0.32 ± 0.232	0.007*	3.1
csuA/B	1 ± 0.243	0.32 ± 0.211	0.002*	3.1
abaI	1 ± 0.244	0.95 ± 0.31	0.232	NA#
A1S_2091	1 ± 0.226	1.2 ± 0.211	0.655	NA
A1S_1510	1 ± 0.254	1.1 ± 0.234	0.215	NA
A1S_0690	1 ± 0.320	1 ± 0.654	0.075	NA
A1S_0114	1 ± 0.182	1.2 ± 0.312	0.512	NA

The expression level of each gene was determined with respect to untreated cells, defined as 1. Values represent means ± SD.

*p values were calculated by student’s *t*-test and values < 0.05 indicated significant difference between treated and untreated groups. #; not applicable refers to genes with no statistically significant difference.

### In vitro infection model for analyzing the antibacterial activity of AgNPs

**Figure 3:**
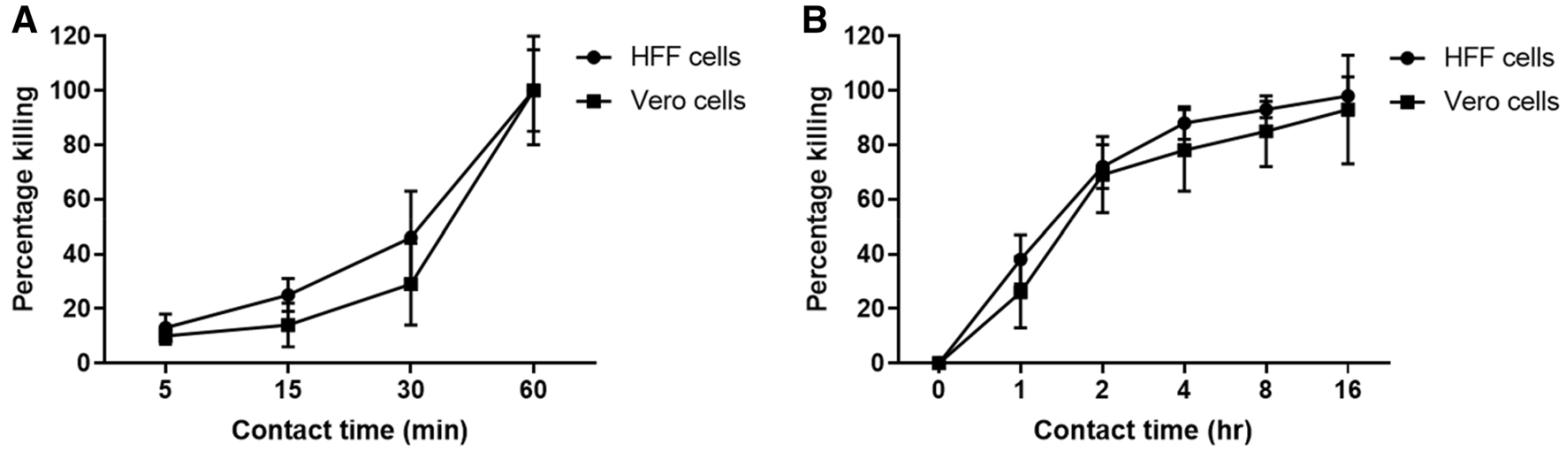
Kinetic profile of the antibacterial activity of AgNPs against extracellular (**A**) and intracellular (**B**) *A. baumannii*. HFF or Vero cells were co-cultured with *A. baumannii* and AgNPs (25 μg/ml) were added. Extracellular killing was evaluated by plating the mixture on Mueller–Hinton agar plates and determination of the CFU, while intracellular killing was evaluated by lysing the cells with Triton X-100 following by culture. The percentage of killing caused by the AgNPs was calculated at the indicated time points relative to the control untreated cells. Experiment was performed in triplicate and the mean ± standard deviation is shown.

An in vitro infection model was conducted to evaluate the antibacterial activity of the synthesized AgNPs against extracellular and intracellular *A. baumannii*. The bacterial killing efficacy increased with increasing the contact time of the treatment. The 25 μg/ml AgNPs displayed strong anti-Acinetobacter activity against extracellular bacteria, showing 10 ± 3% killing efficacy after 5 min and reaching complete killing after 60 min for Vero cells with a similar trend in case of HFF cell line (Fig. 3A). In addition, the killing efficacy against intracellular *A. baumannii* was investigated. There was an increase in killing efficacy with time; the percentage of killed intracellular bacteria was 29 ± 13% after 1 h, which increased to complete killing after 16 h in case of Vero cells and a similar trend was also observed in case of HFF cells (Fig. 3B).

### Evaluation of cytocompatibility of AgNPs on human foreskin fibroblast (HFF-1) and Vero cell lines

**Figure 4:**
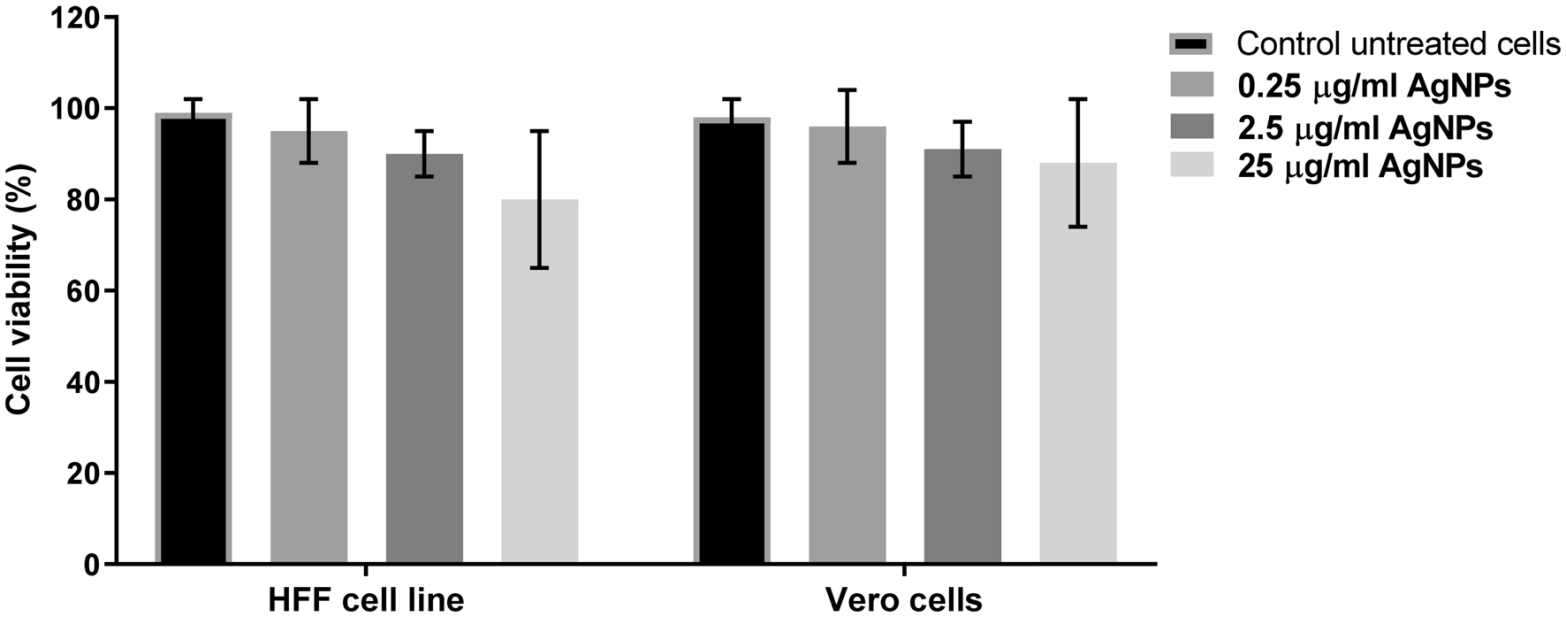
Analysis of in vitro Cytotoxicity of AgNPs. Percentage of the viable HFF and Vero cells treated with different concentrations of AgNPs. Results show the mean ± SEM of three independent experiments.

The biocompatibility of AgNPs was evaluated using the human foreskin fibroblast (HFF) and Vero cell lines. Cell viability test showed that exposure of the human fibroblasts to different AgNPs concentrations, starting from 0.25 to 25 µg/mL (the highest MIC value), did not affect the viability of the cells compared to untreated control cells, indicating that the nanoparticles are not harmful and biocompatible (Fig. 4).

## Discussion

Severe infections associated with the multidrug-resistant bacteria, have increased significantly in the last decades^39–44^. The ability of bacteria to form biofilms is one of the main virulence factors that interferes with antibiotic activity and immune defense response mechanisms^45–48^. *A. baumannii* has developed many virulence factors and is responsible for severe life threatening infections^49–53^. The bacterium affects different sites, including wound infections, pneumonia, UTI. Biofilm formation abilities and various adhesins participate in the pathogenesis of their infections and resistance to antimicrobial drugs^54^. Herein, we observed that the majority of *A. baumannii* were resistant to most of antibiotics and could produce strong biofilms (82.5%). Interestingly, all the isolates which contained *fimH* and *afa/draBC* could produce strong biofilms. *A. baumannii* from wound infections mostly harbored the *fimH*, *afa/draBC*, *cnf1*, *csgA* and *cnf2*^55,56^. It is worth considering that all of these strains were also MDR. The most effective antibiotic against MDR isolates included nitrofurantoin (12%) and imipenem (23%). Our results are in line with other studies which reported a high rate of biofilm formation among MDR-*A. baumannii*^57^.

In the last years new approaches have been developed to enhance the antimicrobial treatment such as the use antibiotic combinations, bacteriophage therapy, usage of antimicrobial NP-based formulations of old antimicrobial agent^58–60^. Recently, several reports have described the improved antimicrobial activity of AgNPs to be used for topical administration against multidrug-resistant bacteria^61^.

The anti-Acinetobater activity of the formulated AgNPs was shown by producing marked zones of inhibition and delay in microbial growth curves. Similar results were reported by previous studies using 14–27 µg/mL AgNPs^62^. The inhibitory activity of AgNPs on Gram-negative bacteria is more efficient than their activity on the Gram-positive bacteria^63,64^. AgNPs exerts potent antibacterial activity through different mechanisms. They have been reported to adsorb in the form of ionic silver (Ag^+^) onto the cytoplasmic membranes of Gram-negative bacteria which leads to destruction of the cell membrane, leakage of bacterial contents and death^65^. In addition, AgNPs also inhibit synthesis of bacterial cell wall^66^. They cause bacterial death through interaction with sulfur groups in essential proteins, generation of reactive oxygen species leading to bacterial death^67^. The high anti-Acinetobacter activity of the AgNPs could be attributed to the small size of the particles and the larger surface area, which allows more contact with the bacterial surface^68^.

Our results showed a potent ability of silver in inhibiting the strong biofilms produced by *A. baumannii.* Also, overnight incubation of sliver to well-formed biofilms lead to their partial removal. This allows the potential use of AgNPs as a biofilm-disrupting agent^69^. Consistent with our results, previous studies have shown efficient effect of AgNPs in inhibiting biofilms produced by different microorganisms^70,71^. Different investigators have evaluated the ability of silver nanoparticles to inhibit biofilm formation by *A. baumannii*, *Pseudomonas aeruginosa* (synergy with tobramycin)*, Staphylococcus aureus* (such as synergy with vancomycin), *Escherichia coli and Klebsiella pneumonia* at similar concentrations^23,72–78^.

Importantly, this is the first report that shows that ability of AgNPs to inhibit the expression of different virulence-related genes (*kpsMII* and *afa/draBC*) and biofilm-related genes (*bap, OmpA, and csuA/B*), which adds to the known mechanism by which silver interferes with microbial growth. We observed that 25 µg/mL of AgNPs significantly decreased the expression of virulence and biofilm-related genes*.*

Studies have shown that the presence of *Csu* gene enables *A. baumannii* strains to attach and form strong biofilms through formation of pili^79^. When *Csu* is inactivated*,* the ability of pili production and subsequently the ability of bacteria to format biofilms is inhibited^80^. Finally, we concluded that decreasing the expression of *bap, OmpA, and csuA/B* by AgNPs is a main approach for decreasing biofilm activity of *A. baumannii* that will help in reducing antibiotic resistance mechanisms^81^. The limitations of our study included lack of an in vivo study, which we plan to study in the future work.

## Conclusion

This study has enhanced our understanding of the characteristics of clinical *A. baumannii* isolates. The analysis of the isolates revealed that most isolates showed high level of resistance to antibiotics, carry bundle of virulence-related genes and different abilities to produce biofilms. Furthermore, treating the bacteria with AgNPs significantly interrupted bacterial growth and multiplication. The activity of AgNPs on *A. baumannii* growth kinetics, biofilm inhibition and dispersion was affected markedly by the strength of the produced biofilms. AgNPs downregulated the transcription level of important virulence and biofilm-related genes. Our findings provide an additional step towards understanding the mechanisms by which AgNPs interfere with the microbial spread and persistence.

## Supplementary information

Supplementary Information 1.

Supplementary Information 2.

## Data Availability

The datasets used and/or analyzed during the current study are available from the corresponding author on reasonable request.
