# Supplementary material for: Antibiofilm and antivirulence potential of silver nanoparticles against multidrug-resistant Acinetobacter baumannii
Source: Sci Rep. 2021 May 24;11:10751. doi: 10.1038/s41598-021-90208-4 (PMC8144575; doi:10.1038/s41598-021-90208-4)
Supplement: Supplementary file 1 — Supplementary Information 1. [file 41598_2021_90208_MOESM1_ESM.docx]

**Title: Antibiofilm and Antivirulence potential of Silver Nanoparticles against Multidrug-Resistant *Acinetobacter baumannii***

**Running Title: Silver Nanoparticles against *Acinetobacter baumannii***

Helal F. Hetta^1&2*^, Israa M.S. Al-Kadmy^3&4*^, Saba Saadoon Khazaal^3^, Suhad Abbas^3^, [Ahmed Suhail](https://www.sciencedirect.com/science/article/pii/S0008622317312861#!)^5^ , Mohamed A. El-mokhtar^1^, Noura H. Abd Ellah^6^ , Esraa A. Ahmed^7,8^, Rasha B. Abd-ellatief ^7^, Eman A. El-Masry^9,10^ , Gaber El-Saber Batiha^11^, Azza A Elkady^12^, Nahed A. Mohamed^13^ and Abdelazeem M. Algammal^14^

^1^Department of Medical Microbiology and Immunology, Faculty of Medicine, Assiut University, Assiut 71515, Egypt.

^2^ Department of Medical Microbiology and Immunology, Faculty of Medicine, Merit University, Sohag, Egypt.

^3^Branch of Biotechnology, Department of Biology, College of Science, AL-Mustansiriyah University, POX 10244, Baghdad-Iraq

^4^Faculty of Science and Engineering, School of Engineering, University of Plymouth, Plymouth, PL4 8AA, UK

^5^Wolfson Nanomaterials & Devices Laboratory, School of Computing, Electronics and Mathematics, Faculty of Science & Engineering, Plymouth University, Devon, PL4 8AA, UK

^6^Department of Pharmaceutics, Faculty of Pharmacy, Assiut University, Assiut, 71526, Egypt.

^7^Department of Pharmacology, Faculty of Medicine, Assiut University, Assiut, 71515, Egypt.

^8^ Centre of Excellence in Environmental Studies (CEES), King Abdulaziz University, Jeddah 21589, Saudi Arabia.

^9^ Microbiology and Immunology Unit, Department of Pathology, College of Medicine, Jouf University, Al-Jouf, Saudi Arabia.

^10^Department of Medical Microbiology and Immunology, College of Medicine, Menoufia University. Egypt.

^11^Department of Pharmacology and Therapeutics, Faculty of Veterinary Medicines, Damanhour University, Damanhur, 22511, Egypt. ^12^Sohag University medical administration, Sohag University, Sohag 82524, Egypt. ^13^ Department of Medical Biochemistry, Faculty of Medicine, Assiut University, Assiut, Egypt. ^14^Department of Bacteriology, Immunology, and Mycology, Faculty of Veterinary Medicine, Suez Canal University, Ismailia, 41522, Egypt***.***

**^*^Corresponding author**

Helal F. Hetta, Email: [helalhetta@aun.edu.eg](mailto:helalhetta@aun.edu.eg); Israa M.S. Al-Kadmy^,^ Email: [**i**sraa.al-kadmy@plymouth.ac.uk](mailto:israa.al-kadmy@plymouth.ac.uk)

**Supplemetal Table1. primer sequences for detection of A baumannii and its virulence-related genes genes.** [**^1-3^**](#_ENREF_1)

| **Gene** | **Primer name** | **Primer Sequence (5'-3')** | **Size of product (bp)** |
| --- | --- | --- | --- |
| ***recA*** | *RecA* F  *RecA* R | CCTGAATCTTCYGGTAAAAC  GTTTCTGGGCTGCCAAACATTAC | 240 |
| ***bla_-_*_oxa51_** | *bla_-_*_oxa51 F_  *bla_-_*_oxa51 R_ | 5'-TAA TGC TTT GATCGG CCT TG-3'  5'-TGG ATT GCA CTT CAT CTT GG-3' | 208 |
| ***afa/draBC*** | afa1  afa2 | GCTGGGCAGCAAACTGATAACTCTC  CATCAAGCTGTTTGTTCGTCCGCCG | 750 |
| ***cnf1*** | cnf1  cnf2 | AAGATGGAGTTTCCTATGCAGGAG  CATTCAGAGTCCTGCCCTCATTATT | 498 |
| ***cnf2*** | cnf2a  cnf2b | AATCTAATTAAAGAGAAC  CATGCTTTGTATATCTA | 543 |
| ***csgA*** | M464  M465 | ACTCTGACTTGACTATTACC  AGATGCAGTCTGGTCAAC | 200 |
| ***cvaC*** | ColV-CF  ColV-CR | CACACACAAACGGGAGCTGTT  CTTCCCGCAGCATAGTTCCAT | 680 |
| ***fimH*** | FimH F  FimH R | TGCAGAACGGATAAGCCGTGG  GCAGTCACCTGCCCTCCGGTA | 508 |
| ***fyuA*** | FyuA f  FyuA R | TGATTAACCCCGCGACGGGAA  CGCAGTAGGCACGATGTTGTA | 880 |
| ***ibeA*** | ibe10 F  fibe10 R | AGGCAGGTGTGCGCCGCGTAC  TGGTGCTCCGGCAAACCATGC | 170 |
| ***iutA*** | AerJ F  AerJ R | GGCTGGACATCATGGGAACTGG  CGTCGGGAACGGGTAGAATCG | 300 |
| ***kpsMT* II** | kpsII F  kpsII R | GCGCATTTGCTGATACTGTTG  CATCCAGACGATAAGCATGAGCA | 272 |
| ***PAI*** | RPAi F  RPAi R | GGACATCCTGTTACAGCGCGCA  TCGCCACCAATCACAGCCGAAC | 930 |
| ***papC*** | pap1  pap2 | GACGGCTGTACTGCAGGGTGTGGCG  ATATCCTTTCTGCAGGGATGCAATA | 328 |
| ***PapG* II, III** | pGf  pGr | CTGTAATTACGGAAGTGATTTCTG  ACTATCCGGCTCCGGATAAACCAT | 1070 |
| ***sfa/focDE*** | sfa1  sfa2 | CTCCGGAGAACTGGGTGCATCTTAC  CGGAGGAGTAATTACAAACCTGGCA | 410 |
| ***traT*** | TraT F  TraT R | GGTGTGGTGCGATGAGCACAG  CACGGTTCAGCCATCCCTGAG | 290 |

**SupplemetalTable 2:primers used for Real time PCR quantification of virulence and biofilm-related genes**

| **Gene** | **Primer sequence:5'-3'** | **Reference** |
| --- | --- | --- |
| ***kpsMII*** | GCGCATTTGCTGATACTGTTG  CATCCAGACGATAAGCATGAGC | [^4^](#_ENREF_4)^,^[^5^](#_ENREF_5) |
| ***afa/draBC*** | ACCCGACGCCGTTTTACATCAACCTG  CCCTTCCCGCCACCTTTCAGCA |  |
| **bap** | AATGCACCGGTACTTGATCC  TATTGC CTGCAGGGTCAGTT | [^6^](#_ENREF_6) |
| ***OmpA*** | CGACGCTTTATCTCTTCG  GGAGCAGCAGGCTTGAAG | [^7^](#_ENREF_7) |
| **abaI** | CCGCCTTCCTCTAGCAGTCA  AAAACCCGCAGCACGTAATAA | [^8^](#_ENREF_8) |
| **csuA/B** | GCAGCTGTTACTGGTCAG  GTCTGTGCGTTCACCACC | [^7^](#_ENREF_7) |
| **A1S_2091** | GTCCACCATCAAATGACAAAGTCC CTGTGTCCTGAATACCTCAGC | [^7^](#_ENREF_7) |
| **A1S_1510** | GATGTTGCTGGTCGTACACC  GACATTGGTAGCTGCACCAG | [^7^](#_ENREF_7) |
| **A1S_0690** | AAACAACCGCAACTCGTGG  CAGCGGCGTCTTTAATACC | [^7^](#_ENREF_7) |
| **A1S_0114** | Gtagagcctgagacgattgatcca  gttggctcaagttctaatttcgtca | [^9^](#_ENREF_9) |
| **16S rRNA** | CTCCTACGGGAGGCAGCAGT  TATTACCG CGGCTGCTGGC | [^10^](#_ENREF_10) |

**Refrences:**

1 Paniagua-Contreras, G. L. *et al.* Comprehensive expression analysis of pathogenicity genes in uropathogenic Escherichia coli strains. *Microbial pathogenesis* **103**, 1-7 (2017).

2 Obata-Yasuoka, M., Ba-Thein, W., Tsukamoto, T., Yoshikawa, H. & Hayashi, H. Vaginal Escherichia coli share common virulence factor profiles, serotypes and phylogeny with other extraintestinal E. coli. *Microbiology* **148**, 2745-2752 (2002).

3 Braun, G. & Vidotto, M. C. Evaluation of adherence, hemagglutination, and presence of genes codifying for virulence factors of Acinetobacter baumannii causing urinary tract infection. *Memórias do Instituto Oswaldo Cruz* **99**, 839-844 (2004).

4 Abdel-Rhman, S. H. Role of Pseudomonas aeruginosa lipopolysaccharides in modulation of biofilm and virulence factors of Enterobacteriaceae. *Annals of microbiology* **69**, 299-305 (2019).

5 Lee, C. S., Wetzel, K., Buckley, T., Wozniak, D. & Lee, J. Rapid and sensitive detection of Pseudomonas aeruginosa in chlorinated water and aerosols targeting gyrB gene using real‐time PCR. *Journal of applied microbiology* **111**, 893-903 (2011).

6 De Gregorio, E. *et al.* Development of a real-time PCR assay for the rapid detection of Acinetobacter baumannii from whole blood samples. *New Microbiol* **38**, 251-257 (2015).

7 Rumbo-Feal, S. *et al.* Contribution of the A. baumannii A1S_0114 gene to the interaction with eukaryotic cells and virulence. *Frontiers in cellular and infection microbiology* **7**, 108 (2017).

8 Lannan, F. M. *et al.* Evaluation of virulence gene expression patterns in Acinetobacter baumannii using quantitative real-time polymerase chain reaction array. *Military medicine* **181**, 1108-1113 (2016).

9 Rumbo-Feal, S. *et al.* Whole transcriptome analysis of Acinetobacter baumannii assessed by RNA-sequencing reveals different mRNA expression profiles in biofilm compared to planktonic cells. *PloS one* **8**, e72968 (2013).

10 Clifford, R. J. *et al.* Detection of bacterial 16S rRNA and identification of four clinically important bacteria by real-time PCR. *PloS one* **7**, e48558 (2012).
